# Quick balance skill improvement after short-term training with error amplification feedback for older adults

**DOI:** 10.1038/s41539-022-00151-w

**Published:** 2023-01-12

**Authors:** Yi-Ching Chen, Gwo-Ching Chang, Wei-Min Huang, Ing-Shiou Hwang

**Affiliations:** 1 Department of Physical Therapy, College of Medical Science and Technology, Chung Shan Medical University, Taichung City, Taiwan; 2 Physical Therapy Room, Chung Shan Medical University Hospital, Taichung City, Taiwan; 3 Department of Information Engineering, I-Shou University, Kaohsiung City, Taiwan; 4 Department of Management Information System, National Chung Cheng University, Chiayi, Taiwan; 5 Department of Physical Therapy, College of Medicine, National Cheng Kung University, Tainan City, Taiwan; 6 Institute of Allied Health Sciences, College of Medicine, National Cheng Kung University, Tainan City, Taiwan

**Keywords:** Motor control, Sensorimotor processing

## Abstract

This study investigated behavioral and cortical mechanisms for short-term postural training with error amplification (EA) feedback in the elderly. Thirty-six elderly subjects (65.7 ± 2.2 years) were grouped (control and EA, *n* = 18) for training in stabilometer balance under visual guidance. During the training session (8 training rounds of 60 s in Day 2), the EA group received visual feedback that magnified errors to twice the real size, whereas the control group received visual feedback that displayed real errors. Scalp EEG and kinematic data of the stabilometer plate and ankle joint were recorded in the pre-test (Day 1) and post-test (Day 3). The EA group (−46.5 ± 4.7%) exhibited greater post-training error reduction than that of the control group (−27.1 ± 4.0%)(*p* = 0.020), together with a greater decline in kinematic coupling between the stabilometer plate and ankle joint (EA: −26.6 ± 4.8%, control: 2.3 ± 8.6%, *p* = 0.023). In contrast to the control group, the EA group manifested greater reductions in mean phase-lag index (PLI) connectivity in the theta (4–7 Hz)(*p* = 0.011) and alpha (8–12 Hz) (*p* = 0.027) bands. Only the EA group showed post-training declines in the mean PLI in the theta and alpha bands. Minimal spanning tree analysis revealed that EA-based training led to increases in the diameter (*p* = 0.002) and average eccentricity (*p* = 0.004) of the theta band for enhanced performance monitoring and reduction in the leaf fraction (*p* = 0.030) of the alpha band for postural response with enhanced automaticity. In conclusion, short-term EA training optimizes balance skill, favoring multi-segment coordination for the elderly, which is linked to more sophisticated error monitoring with less attentive control over the stabilometer stance.

## Introduction

Due to degenerative changes in sensory capabilities and motor systems, the elderly are prone to falls and accidents. The balance problem of older adults can be improved by exercise training to enhance perceptual skills and visuospatial attention. Unlike young adults, older adults tend to adhere more strongly to visual input to control posture^1,2^, notably because they need to minimize sensory ambiguity by down-weighting the more affected non-visual inputs^3^. In this context, a balance-based exergaming system under visual guidance has been developed to improve stance control for the elderly^4,5^. The exergaming system is a motion-tracking device that allows older adults to perform balance exercise by on-line tracking of the body center-of-gravity in a mandatory trajectory under visual guidance^6,7^. A traditional approach is to provide accurate visual feedback, which is believed to effectively add perception–action coupling via frequent self-monitoring^8^.

Nevertheless, accurate visual feedback that displays the real size of task errors (or real task performance) does not necessarily optimize the performance or learning of a visuomotor task. Several behavioral studies have reported that biased visual feedback that displays a worse outcome with error amplification (EA) can yield more positive immediate effects or better training benefits^9–11^. According to the cue utilization hypothesis, EA virtually magnifies visual errors outside the expected state, so the subjects can pay more attention to the unexpected deviant events^12^. Task improvement with EA optimizes behavioral strategies with richer and more frequent corrective attempts to remedy movement deviations^13,14^. An increasing body of neurophysiological evidence supports EA-dependent neuromuscular control in young adults, such as increases in common oscillatory inputs, corticomuscular coherence in the beta band, and discharge irregularity of motor units^9,13^. Nowadays, the EA strategy has been advocated to expedite training in balance control^15,16^ and locomotion stability^17,18^. Given its facilitation effect, the cortical control underlying the improvement in balance skill is still poorly understood, and particularly the plastic functional changes in brain regions vulnerable to aging effects.

If EA works to expedite balance training in older adults, it should provide fast cortical adaptability to balance constraints, overriding the potential negative factors of biased visual feedback that impede learning with aging (such as perceptual conflicts^19^ and declines in resource capacity^20^). Balance training could change one’s postural synergy via whole-brain reorganization^21^. This training involves alterations of the error, sensorimotor, and visual processing, as manifested in changes in local electroencephalography (EEG) or EEG-EEG connectivity in the theta (4–7 Hz), alpha (8–12 Hz), or beta (13–35 Hz) rhythms over the fronto-central and centro-parietal cortical regions^22–24^. For unbiased properties^25^, the minimum spanning tree (MST) is proposed to characterize network characteristics for postural control^26–28^. The MST is the backbone structure of EEG connectivity, mathematically defined as the sub-network connecting all nodes with minimized link weights and without forming loops in the network. EEG networks ranging from star-like to line-like configurations can be parameterized with the MST. MST-based connectomes of older adults performing a postural transfer task revealed that, after stabilometer training, the cortical network was more integrated, with a greater leaf fraction and smaller eccentricity^28^.

The aim of this study was to investigate changes in behavioral strategies and neuro-cortical dynamics following short-term balance training on a stabilometer with visual EA for older adults, with a specific focus on the exploitation of body sway dynamics and inter-regional functional connectivity. It was hypothesized that the training benefits of stabilometer stance with visual EA would be superior to those of traditional visual feedback. Older adults with visual EA could learn how to exploit the ankle strategy better to adapt to fluctuating stabilometer movements. In line with the positive practice effect, a postural EEG network was used to monitor performance and attentional focus with visual EA training by examination of the modulation of the connectivity strength and MST-based network metrics in the theta, alpha, and beta bands.

## Results

### Behavior performance

**Fig. 1:**
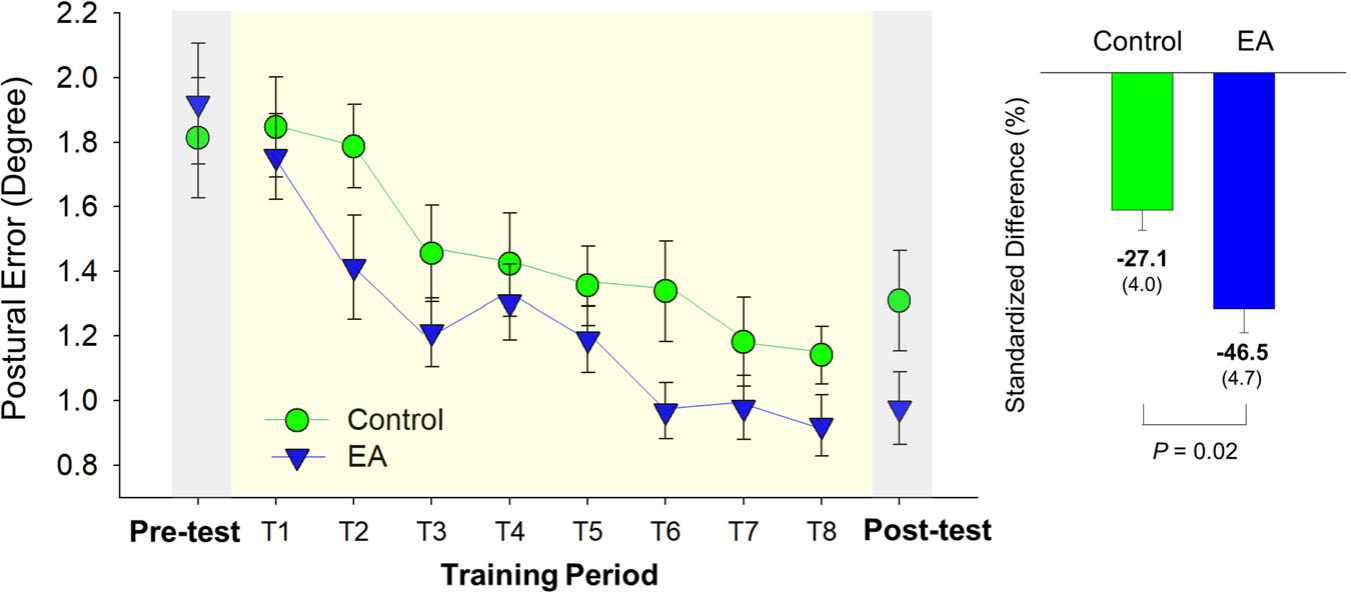
Changes in task errors for the stabilometer task during the practice session for the error amplification (EA) and control groups, in reference to the task errors in the pre-test and post-test. The standardized differences in task errors (or performance benefits) were contrasted between the two groups, in support of a greater amount of balance improvement due to the use of EA feedback. (The error bars on the bar charts represent standard error.).

In reference to postural errors in the pre-test (Day 1) and post-test (Day 3), the left plot of Fig. 1 shows changes in postural errors in the EA and control groups during the training period (Day 2). Postural errors progressively decreased in both groups with practice trials. The results of independent *t* statistics showed better training benefits in the EA group, as indicated by the greater reduction in the standardized differences in postural errors between the pre-test and post-test (*t*_*34*_ = 2.440, *p* = 0.02)(EA: −46.5 ± 4.7%, control: −27.1 ± 4.0%)(Fig. 1, the right plot).

**Fig. 2:**
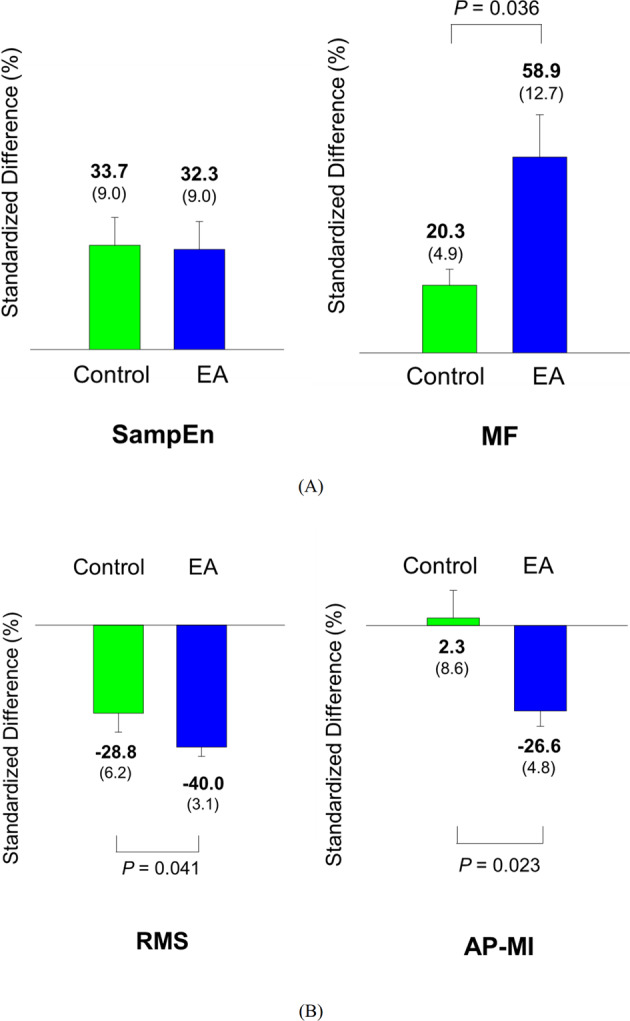
The results of postural fluctuation properties and ankle kinematics properties for the control and EA groups. The contrasts of standardized differences in postural fluctuation properties (sample entropy (SampEn) and mean frequency (MF)(A), and in ankle kinematics properties (root mean square (RMS) and ankle–plate mutual information (AP-MI))(B) between the two groups. (EA: error amplification) (The error bars on the bar charts represent standard error.).

For behavioral strategies of postural control, Table 1 shows the results of Hotelling’s T-squared statistics, which were used to compare the differences in the postural fluctuation variables between the pre-test and post-test for the EA and control groups. The results indicated that postural fluctuation variables were subject to training in both groups (control: Wilks’ Λ = 0.631, *p* = 0.028; EA: Wilks’ Λ = 0.466, *p* = 0.002), with greater sample entropy (SampEn) and mean frequencies (MF) of postural fluctuations in the post-test than in the pre-test (*p* < 0.001 to *p* = 0.021). Figure 2A contrasts the standardized differences in SampEn and MF of postural fluctuations between the EA and control groups. The standardized differences in MF of postural fluctuations of the EA group (EA: 58.9 ± 12.7%, control: 20.3 ± 4.9%) were larger than those of the control group (*t*_34_ = −2.178, *p* = 0.036). In contrast, the standardized differences in the SampEn of postural fluctuations did not differ in the two groups (*t*_34_ = 0.088, *p* = 0.931)(control: 33.7 ± 9.0%, EA: 32.3 ± 9.0%). Table 2 shows the results of Hotelling’s T-squared statistics, which were used to compare the differences in the ankle kinematics (root mean square (RMS) and ankle–plate mutual information (AP-MI)) between the pre-test and post-test for the EA and control groups. The results indicated that ankle kinematics and ankle–plate mutual information of the both groups were subject to training (control: Wilks’ Λ = 0.214, *p* < 0.001; EA: Wilks’ Λ = 0.112, *p* < 0.001). Post-hoc test revealed that RMS (*p* < 0.001) and AP-MI (*p* = 0.002) of ankle kinematics of the EA group decreased with training. But only the RMS (*p* < 0.001) of ankle kinematics of the control group decreased with training. Figure 2B contrasts standardized differences in the RMS of ankle movement and AP-MI between the EA and control groups. The results of independent *t* statistics revealed that the EA group exhibited greater reductions in the standardized differences in the RMS of ankle movement (*t*_34_ = 2.127, *p* = 0.041)(EA: −40.0 ± 3.1%, control: −28.8 ± 6.2%) and AP-MI (*t*_34_ = 2.377, *p* = 0.023)(EA: −26.6 ± 4.8%, control: 2.3 ± 8.6%) than the control group did.

**Table 1:** The comparison of postural fluctuation properties (sample entropy (SampEn) and mean frequency (MF)) between the pre-test and post-test for the error amplification (EA) and control groups.

Postural fluctuations		Pre-test	Post-test	Hotelling’s statistics	Pos hoc test
Control	SampEn	0.194 ± 0.015	0.245 ± .023^a^	Wilks’ Λ = 0.631, *p* = 0.025	*t*_17_ = 2.551, *p* = 0.021, *η*_*p*_^2^ = 0.277
	MF(Hz)	0.310 ± 0.017	0.362 ± .020^a^	*η*_*p*_^2^ = 0.369	*t*_17_ = 3.081, *p* = 0.007, *η*_*p*_^2^ = 0.359
EA	SampEn	0.218 ± 0.024	0.263 ± .023^a^	Wilks’ Λ = 0.466, *p* = 0.002	*t*_17_ = 3.133, *p* = 0.006, *η*_*p*_^2^ = 0.366
	MF(Hz)	0.284 ± 0.024	0.416 ± .030^b^	*η*_*p*_^2^ = 0.534	*t*_17_ = 4.410, *p* < 0.001, *η*_*p*_^2^ = 0.534

^a^Post-test > Pre-test, *p* < 0.05.

^b^Post-test > Pre-test, *p* < 0.001.

**Table 2:** The comparison of ankle kinematics (root mean square (RMS) and ankle–plate mutual information (AP-MI)) between the pre-test and post-test for the error amplification (EA) and control groups.

Ankle kinematics		Pre-test	Post-test	Hotelling’s statistics	Pos-hoc test
Control	RMS (degree)	0.516 ± 0.051	0.475 ± 0.056^b^	Wilks’ Λ = 0.214, *p* < 0.001	*t*_17_ = −7.742, *p* < 0.001, *η*_*p*_^2^ = 0.779
	AP-MI	0.293 ± 0.019	0.283 ± 0.024	*η*_*p*_^2^ = 0.786	*t*_17_ = −0.386, *p* = 0.704, *η*_*p*_^2^ = 0.009
EA	RMS (degree)	0.521 ± 0.064	0.312 ± 0.032^a^	Wilks’ Λ = 0.112, *p* < 0.001	*t*_17_ = −10.641, *p* < 0.001, *η*_*p*_^2^ = 0.869
	AP-MI	0.300 ± 0.020	0.216 ± 0.019^b^	*η*_*p*_^2^ = 0.888	*t*_17_ = −3.700, *p* = 0.002, *η*_*p*_^2^ = 0.446

^a^Post-test < Pre-test, *p* < 0.005.

^b^Post-test < Pre-test, *p* < 0.001.

### EEG connectivity analysis

**Fig. 3:**
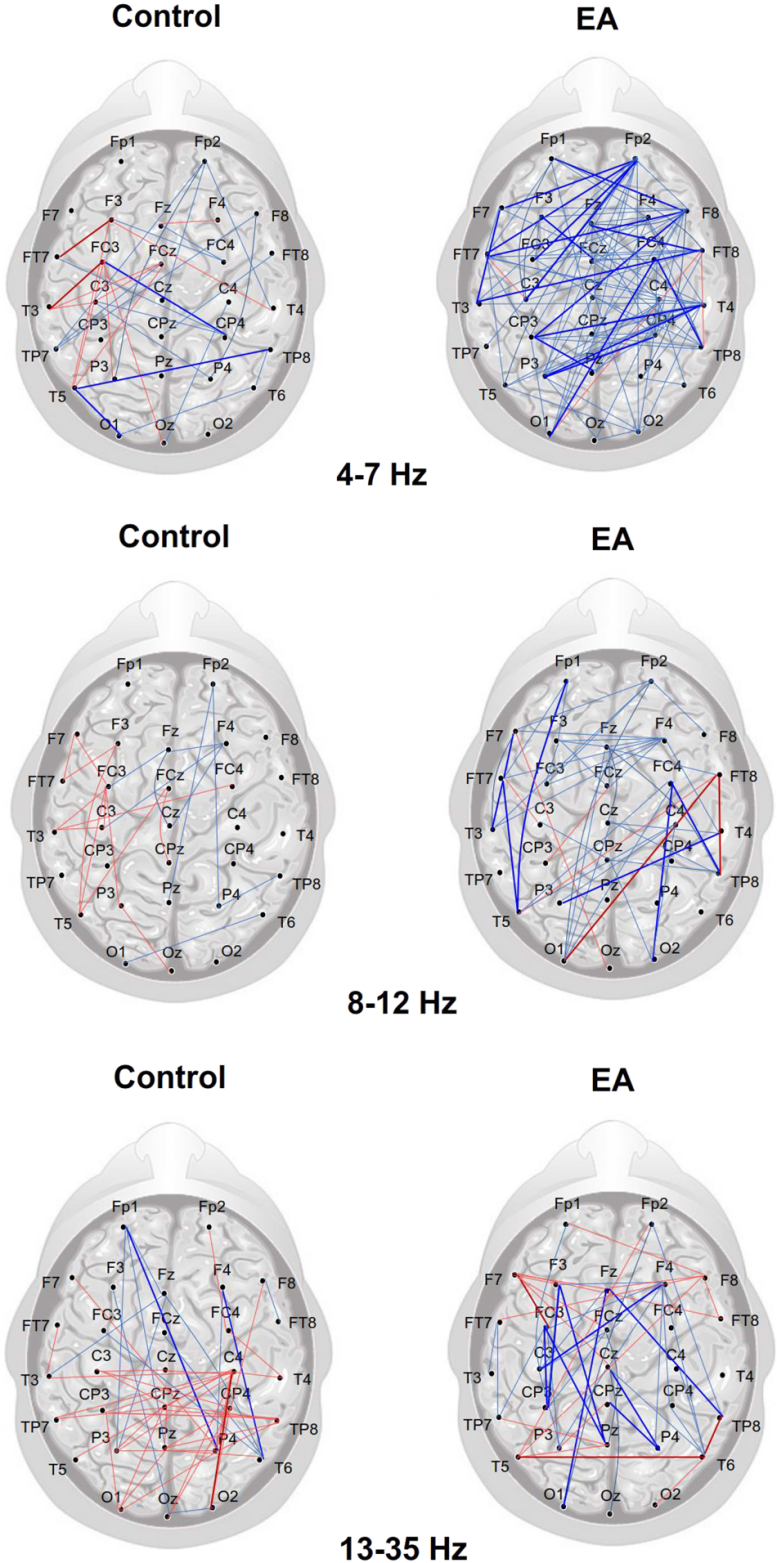
The training-related changes in topology of functional connectivity at theta (4–7 Hz), alpha (8–12 Hz), and beta (13–35 Hz) for the control and EA groups. Greater functional connectivity in the post-test than in the pre-test is labeled with bold (*t*_17_ > 2.898, *p* < 0.005) and light (*t*_17_ > 1.740, *p* < 0.05) red wires. Smaller functional connectivity in the post-test than in the pre-test is labeled with bold (*t*_17_ > −2.898, *p* < 0.005) and light (*t*_17_ > −1.740, *p* < 0.05) blue wires. There are visible differences in training-related functional connectivity between the EA and control groups. In contrast to the control group, the EA group generally exhibited a greater number of changes in supra-threshold connectivity (*p* < 0.005) following postural training, especially for decreases in connectivity strength of the theta and alpha bands in the post-test.

Based on an adjacent matrix of *t* statistics for phase-lag index (PLI) in the pre-test and post-test, Fig. 3 shows training-related differences in the topology of the wiring diagram in various spectral bands for the EA and control groups. The strength of the functional connectivity in the EA group was more susceptible to short-term postural training, showing a greater number of changes in functional connectivity between the pre-test and post-test. Table 3 summarizes the population means of mean phase-lag index (m-PLI) of various EEG spectral bands in the pre-test and post-test of the EA and control groups. The results of Hotelling’s T-squared statistics revealed a significant difference in m-PLI in the EA group (Wilks’ Λ = 0.594, *p* = 0.045) but not in the control group (Wilks’ Λ = 0.873, *p* = 0.551). In the EA group, post hoc analysis further revealed that the m-PLIs of the theta (*t*_17_ = −2.861, *p* = 0.011) and beta (*t*_17_ = −2.424, *p* = 0.027) bands were smaller in the post-test than in the pre-test. Table 4A–C summarize the population means of all the minimum spanning trees (MST) measures at various spectral bands for the EA and control groups. For the control group, all the MST measures in all spectral bands were independent of a training effect ($\left| {t_{17}} \right| = 1.740$, *p* = 0.05). For the EA group, all of the MST measures in the theta (Wilks’ Λ = 0.475, *p* = 0.026) and alpha (Wilks’ Λ = 0.464, *p* = 0.022) bands were significantly different in the pre-test and post-test. For the theta band, post-hoc analysis showed that the diameter was larger in the post-test than in the pre-test (*t*_17_ = 3.617, *p* = 0.002), whereas the average eccentricity was greater in the post-test than in the pre-test (*t*_17_ = 3.315, *p* = 0.004). For the alpha band, the leaf fraction was smaller in the post-test than in the pre-test (*t*_17_ = −2.638, *p* = 0.030). The changes in MST measures indicated more line-like EEG networks in the theta and alpha bands after visual EA training.

**Table 3:** The comparison of mean inter-regional connectivity strength (mean phase-lag index (m-PLI)) between the pre-test and post-test for the error amplification (EA) and control groups.

m-PLI		Pre-test	Post-test	Hotelling’s statistics	Pos hoc test
Control	Theta (4–7 Hz)	0.163 ± 0.001	0.162 ± 0.002	Wilks’ Λ = 0.873, *p* = 0.551	
	Alpha (8–12 Hz)	0.135 ± 0.001	0.136 ± 0.002	*η*_*p*_^2^ = 0.127	
	Beta (13–35 Hz)	0.116 ± 0.004	0.117 ± 0.001		
EA	Theta (4–7 Hz)	0.165 ± 0.001	0.158 ± 0.002^a^	Wilks’ Λ = 0.594, *p* = 0.045	*t*_17_ = −2.861, *p* = 0.011, *η*_*p*_^2^ = 0.325
	Alpha (8–12 Hz)	0.137 ± 0.002	0.132 ± 0.001^a^	*η*_*p*_^2^ = 0.406	*t*_17_ = −2.424, *p* = 0.027, *η*_*p*_^2^ = 0.257
	Beta (13–35 Hz)	0.118 ± 0.001	0.117 ± 0.001		*t*_17_ = −1.795, *p* = 0.090, *η*_*p*_^2^ = 0.159

^a^Post-test < Pre-test, *p* < 0.05.

**Table 4:** The comparison of network graph measures at different spectral bands between the pre-test and post-test for the error amplification (EA) and control groups.

(A)					
Theta (4–7 Hz)		Pre-test	Post-test	Hotelling’s statistics	Pos hoc test
Control	Leaf Fraction	0.564 ± 0.003	0.569 ± 0.003	Wilks’ Λ = 0.641, *p* = 0.156 *η*_*p*_^2^ = 0.359	
	Diameter	0.334 ± 0.001	0.331 ± 0.002		
	Ave. eccentricity	0.263 ± 0.001	0.262 ± 0.001		
	BC_max_	0.725 ± 0.002	0.727 ± 0.002		
EA	Leaf fraction	0.565 ± 0.002	0.562 ± 0.003	Wilks’ Λ = 0.475, *p* = 0.026 *η*_*p*_^2^ = 0.525	*t*_17_ = −1.084, *p* = 0.293, *η*_*p*_^2^ = 0.065
	Diameter	0.332 ± 0.002	0.339 ± 0.002^a^		*t*_17_ = 3.617, *p* = 0.002, *η*_*p*_^2^ = 0.435
	Ave. eccentricity	0.263 ± 0.001	0.267 ± 0.001^a^		*t*_17_ = 3.315, *p* = 0.004, *η*_*p*_^2^ = 0.393
	BC_max_	0.726 ± 0.002	0.724 ± 0.002		*t*_17_ = −0.584, *p* = 0.567, *η*_*p*_^2^ = .020
(B)					
Alpha (8–12 Hz)		Pre-test	Post-test	Hotelling’s statistics	Pos hoc test
Control	Leaf fraction	0.567 ± 0.003	0.570 ± 0.003	Wilks’ Λ = 0.719, *p* = 0.295 *η*_*p*_^2^ = 0.281	
	Diameter	0.332 ± 0.001	0.329 ± 0.002		
	Ave. eccentricity	0.262 ± 0.001	0.259 ± 0.001		
	BC_max_	0.726 ± 0.002	0.733 ± 0.002		
EA	Leaf fraction	0.562 ± 0.003	0.534 ± 0.003^b^	Wilks’ Λ = 0.464 *p* = 0.022 *η*_*p*_^2^ = 0.536	*t*_17_ = −2.638, *p* = 0.030, *η*_*p*_^2^ = 0.248
	Diameter	0.335 ± 0.001	0.336 ± 0.001		*t*_17_ = 0.777, *p* = 0.448, *η*_*p*_^2^ = 0.034
	Ave. eccentricity	0.264 ± 0.001	0.266 ± 0.001		*t*_17_ = 1.647, *p* = 0.118, *η*_*p*_^2^ = 0.138
	BC_max_	0.728 ± 0.002	0.724 ± 0.002		*t*_17_ = -1.641, *p* = 0.119, *η*_*p*_^2^ = 0.137
(C)					
Beta (13–35 Hz)		Pre-test	Post-test	Hotelling’s statistics	Pos hoc test
Control	Leaf fraction	0.573 ± 0.006	0.570 ± 0.005	Wilks’ Λ = 0.821, *p* = 0.567 *η*_*p*_^2^ = 0.179	
	Diameter	0.327 ± 0.004	0.331 ± 0.003		
	Ave. eccentricity	0.259 ± 0.003	0.262 ± 0.002		
	BC_max_	0.731 ± 0.004	0.727 ± 0.003		
EA	Leaf fraction	0.569 ± 0.003	0.570 ± 0.006	Wilks’ Λ = 0.736, *p* = 0.333 *η*_*p*_^2^ = 0.264	
	Diameter	0.323 ± 0.003	0.329 ± 0.004		
	Ave. eccentricity	0.263 ± 0.002	0.260 ± 0.003		
	BC_max_	0.732 ± 0.003	0.729 ± 0.004		

(A) theta band (4–7 Hz), (B) alpha band (8–13 Hz), (C) beta band (13–35 Hz). (BCmax: maximal betweenness centrality).

^a^Post-test > Pre-test, *p* < 0.005.

^b^Post-test < Pre-test, *p* < 0.05.

## Discussion

The EA and control groups consistently showed training benefits with significant error reduction in the stabilometer postural task. However, the EA group demonstrated more remarkable task improvement in error reduction (−46.5 ± 4.7%) than that of the control group (−27.1 ± 4.0%). Our finding in physically-active older adults replicated previous behavioral studies that reported a superior training effect of EA on stance^15,16,29^ and gait-like activity^30^ in young adults. The novel finding in this study is the new understanding of the distinct behavioral strategies and cortical reorganization resulting from EA-based training. At the behavioral level, the trained EA group exhibited a greater increase in the mean frequency of postural fluctuations and kinematic uncoupling of the plate and ankle movements. At the cortical level, the trained EA group manifested greater declines in the mean strength of EEG–EEG connectivity of the theta and alpha bands, which were not evident in the control group. With MST analysis, the EA group was noted to exhibit differences in EEG–EEG backbone connectivity in the theta (greater diameter and average eccentricity) and alpha (smaller leaf fraction) bands, which were also absent in the control group.

The older adults were trained with stabilometer stance, which is commonly used to assess dynamic balance control in rehabilitation clinics. The ankle joint plays the most important role in compensating for body shifts by tuning the contact point of the stabilometer and floor along with the projected line of the center of gravity^31^. In this study, high kinematic coupling between plate and ankle movements explained the reliance on the ankle strategy for stabilometer stance^32,33^. With traditional visual feedback, the trained control group demonstrated smaller ankle fluctuation movement without significantly changing the ankle–plate kinematic coupling in the post-test (Table 2). This fact implied that the control group learned to stabilize the stabilometer stance with mastery over the ankle strategy. In contrast, the trained EA group attained the same postural goal with less reliance on the ankle strategy, as the trained EA group not only decoupled the ankle–plate kinematic coupling but also reduced the ankle fluctuation movements (Table 2). Although we did not directly measure the joint kinematics of the whole body, the EA-related postural improvements with de-emphasis of the ankle strategy presumably resulted from the use of biomechanical degrees of freedom from other body portions (such as the knee, hip, and trunk)^34,35^. The idea of freeing the degrees of freedom with synergy changes at the later stage of motor learning was proposed by Bernstein (1967)^36^. In addition, the EA group demonstrated a higher mean frequency in post-trained postural fluctuations (Table 1, Fig. 2A, the right plot). The learning-related increase in the mean frequency of postural fluctuations implies that postural control after visual EA training relied less on visual information, acknowledging that the postural response consisted of fewer low-frequency postural components predominantly linked to visual control^37^. Along with the smaller size of postural fluctuations, the increases in the sample entropy of the postural fluctuations in the post-test indicates that postural control became more complex, skillful, and automatic after the training with visual EA^38,39^. When a healthy subject was trained with concurrent visual feedback of center of pressure, the complexity of postural sway increased over the whole course of the skill acquisition phase^38^. In a related study, athletes with greater gymnastic skills exhibited higher postural stability and complexity than did athletes with lower gymnastic skills^39^.

The control and EA groups both demonstrated visible changes in cortical plasticity in the post-test, as indicated by post-training variations in EEG–EEG functional connectivity (Fig. 3). However, to be strict, the post-training changes in functional connectivity were relatively weaker (less significant) in the control group than in the EA group. The EA group demonstrated a greater number of training-related changes in suprathreshold connectivity (*p* < 0.005)(Fig. 3) with significant decreases in the mean PLI (m-PLI) of the theta (4–7 Hz) and alpha (8–12 Hz) bands in the post-test (Table 3). In contrast, the m-PLI of the control group did not vary with training.

The theta rhythm functionally serves the functions of movement exploration^40^, performance monitoring^41^, and selective release of motor inhibition^42^. The theta rhythm, especially in the mid-frontal area, potentiates due to unexpected outcomes and task errors^43^. Cortical activities across different areas are tuned to large-amplitude theta oscillation^44^, enabling the brain to minimize free energy due to errors with integrated information^45^. In a postural task, theta connectivity may signify neurocognitive processes to plan corrective steps and/or analyze falling incidence^46^. Postural destabilization, such as during unipedal stance or sudden postural perturbation^23^, is associated with a higher level of mid-frontal theta activity^47,48^. In the EA group, the training-dependent decline in theta connectivity during stabilometer stance was behaviorally contingent on performance improvements with relatively smaller error sizes in the post-test (Table 3) (Fig. 3, the upper right plot). In proportion to the error size, theta activity peaked in the initial stage of learning, but it declined after sophisticated learning associations were built. A training-dependent decline in theta activity in the EA group was related to error contexts for disengagement of the frontal executive functions^49,50^. In effect, frontal disengagement in motor performance is also seen during late adaptation to a force field^51^ and to visuomotor rotation in young adults^52^. In contrast, the control group, who received traditional visual feedback, tended to show less frontal deactivation with training (Fig. 3, the upper left plot). A higher level of frontal theta power to monitor performance is a less efficient mechanism to consolidate motor memory in older adults with a greater extent of structural decline^53^. The use of MST to analyze the theta backbone network revealed that only the network configuration of the EA group was altered, with increases in diameter and average eccentricity in the post-test (Table 4A). Hence, the theta MST network of the trained EA group shifted towards a more ordered and decentralized configuration; this shift was similar to that commonly found in older children as compared with their younger counterparts^54^. It is likely that the EA group developed a more elaborate cortical network for performance monitoring, which contributed to sensorimotor confidence under the condition of smaller postural errors^55^.

According to the cortical idling hypothesis, synchronization of alpha activity reflects cognitive inactivity and/or inhibition of task-irrelevant information^56,57^. The practice of coordinated movement can lead to decreases in the coherence of the bilateral primary motor/sensorimotor areas in the alpha band^58,59^. The practice-induced decline in alpha-band connectivity was consequent to performance improvement. Moreover, a recent fMRI work of Schubert et al. (2021) revealed a causal role of alpha oscillations in the gating of information transfer within a cortico-cerebellar network during the learning process of sequential finger movements^60^. When the sequential movements become progressively skilled and are performed more automatically, alpha oscillations within a cortico-cerebellar network are decoupled, leading to a decrease in cognitive demand. The connectivity scenarios are consistent with the post-test suppression of m-PLI in the alpha band with visual EA found in this study (Table 3). The weaker alpha connectivity might also explain the automatic postural control after training with visual EA, as manifested in the smaller postural fluctuations and higher complexity in the post-test (Table 1). In addition, analysis of the MST-based network in the alpha band revealed feedback-dependent neuroplastic changes in the backbone network. Only the EA group exhibited a decrease in the leaf fraction after postural training with visual EA (Table 4B), in support of a more line-like organization of the alpha network. Although little known is about the relation between motor performance and the line-like organization of the alpha network, a low leaf fraction of the MST-based alpha network has been noted in children with autism spectrum disorder^61^ and in patients with mild cognitive impairment due to Alzheimer’s disease in the resting state with eye closure^62^. By analogy, the low leaf fraction of the alpha MST-based network in this study would suggest the spatial spread of cortical idling for information flow in the thalamo-cortical system^61,62^. This occurred because the older adults, having better balance skill after the EA training, needed less attentive capture of visual cues to refine posture.

As occurs in healthy young adults^63^, short-term training with visual EA has the potential to leverage the principle of neuroplasticity to expedite the achievement of postural training goals for healthy older adults. In practice, visual EA can be easily delivered via virtual reality (VR) technology, which can provide flexible manipulation of the postural error size within an immersive gaming environment. The combined approach is a promising intervention to improve postural balance in the rehabilitation realm. However, any manipulation of the error gain of visual EA should be done pragmatically. As visual EA is expected to enhance visual-spatial attention during training, it could simultaneously increase the cognitive load and performance stress during postural training^20^. In addition, excessively large error amplification could enhance perceptual conflicts among the visual, vestibular and proprioception inputs, with the result that the subjects could fail to react to performance loss and realize the error causes^13^. Hence, caution must be taken to select appropriate error gain to minimize the potential disadvantages associated with visual EA. Finally, the relatively limited number of subjects might have led to sampling bias, which suggests the need for a greater population size for further investigation. Despite this fact, this study reveals an important implication of virtually augmented errors for VR technology.

Our findings may have substantial implications for the optimization of balance practice with an exergaming system, particularly in older adults. Short-term visual EA feedback provides superior training benefits for stabilometer stance as compared with those of traditional visual feedback. With visual EA, the trained older adults relied less on the ankle strategy to stabilize the stabilometer stance. Motor networks were contextually reorganized with the size of visualized errors, with more pronounced and widespread neuroplastic changes in the EA group. Changes in MST-based network metrics suggest that older adults trained with visual EA have an increased ability of performance monitoring, with less attentional focus on improved stabilometer stance. The behavioral and neural scenarios suggest that EA visual feedback has the potential to be combined with a balance-based exergaming system to expedite the training effect in older adults.

## Methods

### Ethics

Participants were monetarily compensated for the duration of the experiment and provided written informed consent to take part in the study, in accordance with the Declaration of Helsinki. This study was approved by an authorized institutional human research review board at the University Hospital (No. B-ER-105-032).

### Participants

A total of 36 participants (20 females and 16 males; age: 61–74 years, mean = 65.9 ± 2.2 years) were recruited in this study and provided written consent. None of the participants were receiving medication for neurological or orthopedic problems. All participants had corrected-to-normal eyesight. They were physically active with regular exercise habits of roughly 1 h per day.

### Experimental procedures and instrumentation

**Fig. 4:**
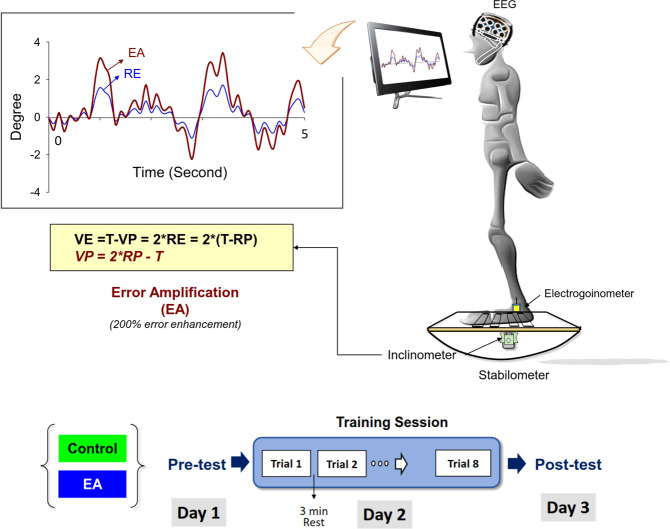
System setup and experimental training protocol. The participants coupled the line of visualized postural sway (VP) to the target line (T) under the guidance of online visual feedback. Balance performance of the trained stabilometer task was measured in the pre-test (Day1) and post-test (Day 3). During the practice session (Day 2), the participants in the control group were trained with traditional visual feedback that displayed visualized errors (VE) equivalent to real errors (RE) in the computer monitor at eye level (VE = RE). By real-time mathematical transformation, the error amplification (EA) group were trained with visual feedback that virtually double the real errors (RE) during the training session (VE = 2*RE). (EA: error amplification, RP: real postural sway).

This study used a randomized, repeated measures, between-groups, parallel design. The participants were randomly assigned to two groups, the control (11 females and 7 males; age: 63–71 years, mean = 65.7 ± 2.2 years) and error amplification (EA) (9 females and 9 males; age: 61–74 years, mean = 66.2 ± 2.6 years) groups. The participants visited the laboratory on three consecutive days (Fig. 4). Three trials of baseline postural performance of stabilometer stance were measured in the pre-test (Day 1). The trials were interleaved with rest periods of 3 min. The participants were asked to maintain upright stance as steadily as possible for 60 seconds on a 50 cm × 58 cm stabilometer (radius: 25 cm; height: 18.5 cm) under visual guidance. While doing so, they carefully coupled a line representing the plate movement of the stabilometer to a horizontal target line (the ground level), simultaneously displayed on a computer monitor in front of the subjects at eye level (Fig. 4). During the training session (Day 2), there were 8 training rounds of 60 s on the stabilometer, interleaved with rest periods of 3 min. The subjects in the control group were provided with traditional visual feedback to train stabilometer stance (the same as Day 1). The visual feedback displayed the real trajectory of the plate movement on the monitor such that the control group perceived real errors (RE) during postural training. On the other hand, the subjects in the EA group were trained with visual feedback of virtual plate trajectory via mathematical transformation that doubled the size of execution errors (Fig. 4). The behavioral paradigm of error amplification was similar to that of a previous study by Hwang et al. (2017)^9^, which led to an immediate positive effect on manual tracking. Briefly, virtual error amplification in visual feedback was achieved by real-time mathematical transformation of the trajectory of the stabilometer plate movement. For the EA group during the training session, the visualized plate movement (VP) on the monitor was not the real trajectory of the stabilometer plate movement. Instead, the VP was manipulated with a formula (VP = 2*RP-T, RP: real plate movement, T: target signal). Hence, the size of the visualized error (VE) that the EA group perceived during the training session was twice that of the real error (VE = 2*RE)^9,14^. Three trials of postural performance of the stabilometer stance after short-term training were measured in the post-test (Day 3). The experiment trials in the post-test were performed identically to those in the pre-test. Stabilometer stance performance for both the EA training and control groups was measured under the visual guidance of real error feedback.

### Experimental setup

Two kinematic data, namely, the tilting angle of the stabilometer plate and the angular displacement of the ankle joint, were collected. During the pre-test, training session, and post-test, an inclinometer (Model FAS-A, LORD MicroStrain, USA) mounted on the center of the stabilometer was used to register variations in the tilting angle of the stabilometer plate. Angular movement of the ankle in the sagittal plane (plantarflexion/dorsiflexion) was recorded with electrogoniometers (Model SG110/A, Biometrics Ltd, UK). Distal and proximal sensors were placed along the lateral aspects of the 5th lateral malleolus and the fibular bone, respectively. The ankle angular movement was measured because postural control on a stabilometer relies predominantly on the ankle strategy^31–33^. In the pre-test and post-test, a NuAmps amplifier (NeuroScan Inc., EI Paso, USA) and Ag–AgCl electrodes were used to measured cortical activities (scalp EEG) that synchronized with kinematic data during the stabilometer stance. According to the International 10–20 system, scalp EEG signals were localized at different cortical areas (Fp_1/2_, F_z_, F_3/4_, F_7/8_, FT_7/8_, FC_z_, FC_3/4_, C_z_, C_3/4_, CP_z_, CP_3/4_, P_z_, P_3/4_, T_3/4_, T_5/6_, TP_7/8_, O_z_, and O_1/2_). Reference electrodes were placed on each side of the mastoid process (A_1_/A_2_), and the ground electrode was placed on the forehead. Two electrooculography (EOG) electrodes were placed infra- and supra-orbitally at the right eye for subtraction of eye movement and blink artifacts. The impedances of all the electrodes were below 5 kΩ and were referenced to linked mastoids of both sides. The EEG data were recorded by setting a band-pass filter (cut-off frequencies: 0.1–70 Hz) and a 60 Hz notch filter. The EEG data and the angular plate movement were integrated and synchronized by the AD controller of the LabView platform (Labview v.8.5, National Instruments, USA). The sampling rate for kinematic data and EEG was set at 1 kHz.

### Data analysis

The positional trajectory of the stabilometer plate and ankle angular movement were conditioned with a 4th-order low-pass Butterworth filter (cutoff frequency: 6 Hz). The positional trajectory of the stabilometer plate and ankle angular movement of the first and last 2 s were not analyzed to ensure data stability. After that, the sizes of postural errors in the stabilometer stance in the pre-test, training sessions, and post-test were represented by the root mean square (RMS) of mismatches between the positional trajectory of the stabilometer plate and the horizontal target line. The training benefits of the stabilometer task were indexed with the standardized difference in task errors between the pre-test and post-test ((post-test − pre-test)/pre-test*100%). To feature postural strategies, the mean frequency (MF) and sample entropy (SampEn) of postural fluctuations (positional trajectory after removal of linear trends) were estimated. The mean frequencies (MF) of the postural fluctuations were estimated with the power spectra of the postural data, estimated using a fast Fourier transform and the Welch method (Hanning window, window length: 15 s, overlapping time segment: 25% × window length) with a spectral resolution of 0.02 Hz. The MF represented a spectral shift in postural sway and responsiveness for postural regulation. The postural fluctuations were first down-sampled to 100 Hz prior to sample entropy calculation. The mathematical formula for sample entropy was

1 $${\rm{SampEn}}\left( {m,r,N} \right) = - \log \left(\mathop {\sum}\limits_{i = 1}^{N - m} {A_i} /\mathop {\sum}\limits_{i = 1}^{N - m} {B_i}\right ),$$

where *r* = 15% of the standard deviation of the data, *m* is the length of the template (*m* = 2), and *N* is the number of data points in the time series. *A*_*i*_ is the number of matches of the *i*th template of length *m* + 1 data points, and *B*_*i*_ is the number of matches of the *i*th template of length *m* data points. Postural sway regularity reflects the degree of attentional investment in postural control^64^. An increase in the regularity (or smaller SampEn) of postural fluctuations indicates a higher degree of attentional involvement on postural response. The amount of ankle angular movement was indexed with RMS. The degree of kinematic coupling between ankle and plate movements was determined with mutual information (AP-MI)^32,33^. Mutual information (*MI*(*X; Y*)) ($MI\left( {X;Y} \right)$) was defined as

2 $$MI\left( {X;Y} \right) = \mathop {\sum}\limits_{y \in Y} {\mathop {\sum}\limits_{x \in X} {p\left( {x,y} \right)\log } } (p(x,y)/p_1\left( x \right)p_2(y)),$$

where *p*(*x*, *y*) is the joint probability density function of detrended ankle angular movement (*X*) and stabilometer plate movement (*Y*), and *p*_*1*_(x) and *p*_*2*_(y) are the marginal probability density functions of the two time series, respectively. The higher AP-MI indicated that a greater portion of postural fluctuations could be accounted for by ankle angular movement, or a greater reliance on the ankle strategy. All of the behavioral variables of the three trials in the pre-test and post-test were averaged for each participant.

**Fig. 5:**
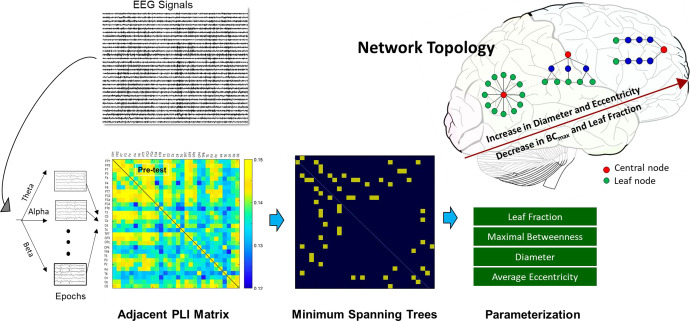
Schematic representation of network properties with EEG-based minimum spanning trees (MSTs). A functional connectivity matrix of the preprocessed sub-band EEG (theta, alpha, or beta rhythms) was constructed using the phase-lag index (PLI) (from zero to one). Four important variables (leaf fraction, maximal betweenness centrality (BC_max_), diameter, and average eccentricity) were used to index brain network organization.

To estimate the inter-regional functional connectivity, EEG data collected during the stabilometer stance in the pre-test and post-test were analyzed. All the EEG data were first filtered between 1 and 60 Hz using a zero-phase finite impulse response (FIR) filter (60 dB/octave) to remove the DC shift. To perform EEG correction of ocular artifacts with regression analysis^65^, eye blinks were detected by creating a bipolar vertical EOG channel in the infraorbitally-placed electrode and the superorbitally-placed electrode of the right eye. Eye blinks in the EEG of an experimental trial were first corrected with the NeuroScan 4.3 software program (NeuroScan Inc., EI Paso, TX, USA). Then only EEG data from the 3rd to 58th seconds of the run were segmented into 2 s epochs. All computations were performed on individual EEG epochs and visually rechecked by researchers to preclude the EEG epochs containing visible eye blinks. The cleaned data for each subject were bandpass filtered into three bands, including the theta (4–7 Hz), alpha (8–12 Hz), and beta (13–35 Hz) bands. Then the phase-lag index (PLI) between the EEG signals of all EEG electrode pairs and the variables of minimum spanning trees (MST) for each sub-band EEG in an epoch were calculated in the pre-test and post-test (Fig. 5). Oscillations under 4 Hz were not analyzed because they were easily contaminated by unexpected movement artifacts^66^. The PLI indexes the distribution asymmetry of the phase differences in the instantaneous phases of two time series were derived from the Hilbert transformation. If $\varphi (t)$ is the phase difference, the PLI is defined as

3 $${\rm{PLI}} = \left| {E\left\{ {{\it{\rm{sgn}}}(\Delta \varphi (t))} \right\}} \right|,$$

where sgn is a function that extracts the sign of a real number. *E* stands for the expected value. The PLI ranged from 0 to 1. A low PLI reflects continuous and uniform information flow between the electrode pairs, as compared with a high PLI, which implies dynamic information flow with high irregularity between the electrode pairs^27^. Compared to different connectivity measures, the advantage of PLI is that it is relatively immune to noise and volume conduction for EEG measures^27,67^.

Based on the PLI adjacent matrix, the backbone structure of EEG functional connectivity was further characterized with minimum spanning trees (MSTs)^26–28^ (Fig. 5). For each spectral sub-band, four key graph measures (leaf fraction, diameter, average eccentricity, and maximal betweenness centrality (BC_max_)) were used to characterize the EEG MST network^26,66^. A more integrated network has a higher BC_max_/leaf fraction and lower diameter/average eccentricity^66^. These MST variables of the three experimental trials were averaged across subjects in each group. Signal processing of the EEG data was performed in Matlab (Mathworks Inc. Natick, USA). The PLI values of the EEG electrode pairs were calculated with the functions of HERMES for Matlab^68^. The parameterization of network properties from MST was accomplished with functions of the Brain Connectivity Toolbox^69^.

### Statistical analysis

**Fig. 6:**
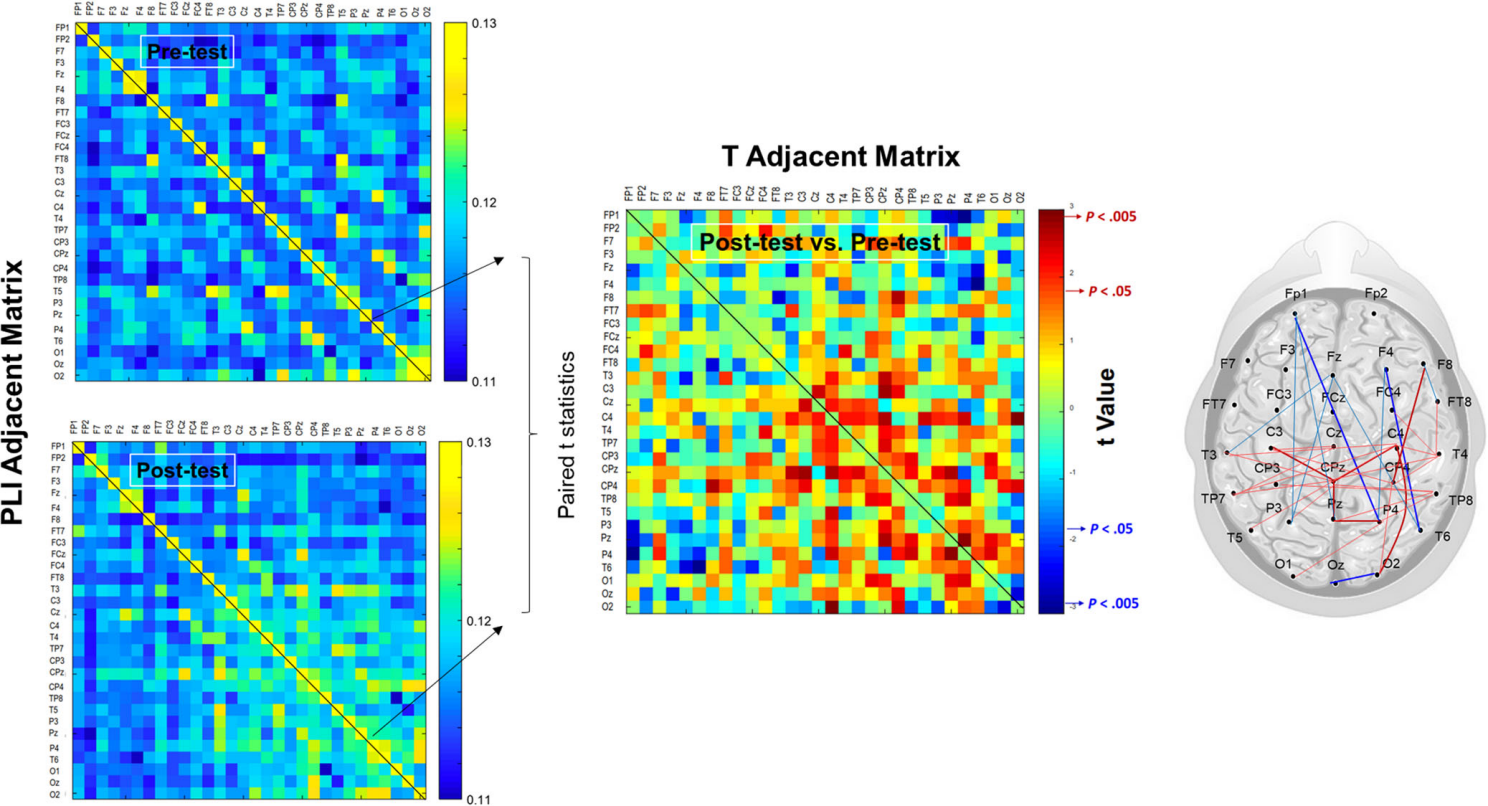
Acquisition of the adjacent matrix of *t*-values that represent training effect on functional connectivity by contrasting the phase-lag index (PLI) of electrode pairs between the pre-test and post-test conditions with paired t statistics. Supra-threshold connectivity was denoted as a significant difference in PLI between the pre-test and post-test (*p* < 0.005).

The primary aim of this study was to contrast training benefits and corresponding cortical adaptations for stabilometer stance after short-term postural training with traditional visual feedback (control) and error amplification (EA) feedback. Independent *t* statistics were used to compare standardized differences in postural errors ((post-test − pre-test)/pre-test) between the control and EA groups. In terms of the PLI of an electrode pair, training-related differences in inter-regional connectivity of the three sub-bands (theta, alpha, and beta) were contrasted with paired *t* statistics for the control and EA groups (Fig. 6). Multi-variate Hotelling’s T-squared statistics were used to compare differences in the postural fluctuation variables (SampEn and MF) and ankle kinematic data (RMS and ankle–plate coupling) of the stabilometer task between the pre-test and post-test for the control and EA groups. Likewise, Hotelling’s T-squared statistics were used to examine the mean PLI and all MST variables of the three sub-bands (theta, alpha, and beta) between the pre-test and post-test in the two groups. The post-hoc test was the Simes test, which, unlike the Bonferroni test, would not produce over-correction. For all post hoc hypotheses ($H = \cap _{i = 1}^m$), the Simes test did not reject elementary *H*_*i*_ if *p*_*i*_ ≤ i*0.05/m for ordered unadjusted *p* values (*p*_*1*_ ≤ ... ≤ *p*_*m*_). The type 1 error rate using the Simes test proved to be exactly 0.05. The effect size was determined by partial eta squared (*η*_*p*_^2^). Data are presented as group means ± standard errors. All statistical analyses were performed in IBM SPSS Statistics (v19). The level of significance was 0.05.

### Reporting summary

Further information on research design is available in the Nature Research Reporting Summary linked to this article.

## Supplementary information

Reporting Summary

## Data Availability

Anonymized raw EEG and behavioral data of the participants who have given (anonymized) data sharing consent are available from the corresponding author upon reasonable request.

## Code availability

Enquiries about code access can be made to the corresponding author.
